# Role of miRNAs in Regulating Ascending Aortic Dilation in Bicuspid Aortic Valve Patients Operated for Aortic Stenosis

**DOI:** 10.3390/ijms26020779

**Published:** 2025-01-17

**Authors:** Antonio de Jesús Sanchez-Garcia, Mauricio Soule-Egea, Giovanny Fuentevilla-Alvarez, Gilberto Vargas-Alarcon, Benjamín Iván Hernández-Mejia, Humberto Martínez-Hernández, Sergio Luis Mora-Canela, Felipe Santibanez-Escobar, Valeria Ávila-Martinez, Vicente Castrejón-Tellez, Edith Alvarez-León, Regina de la Mora-Cervantes, Israel Pérez-Torres, María Elena Soto

**Affiliations:** 1 Cardiothoracic Surgery Department, Instituto Nacional de Cardiología Ignacio Chávez, Juan Badiano No. 1, Col. Sección XVI, Mexico City 14080, Mexico; ajbsbl@hotmail.com (A.d.J.S.-G.); mauricio.soule@cardiologia.org.mx (M.S.-E.); ihm56@hotmail.com (B.I.H.-M.); humbertomartinez@hotmail.com (H.M.-H.); sergiocanelamd@gmail.com (S.L.M.-C.); santi_f@yahoo.com (F.S.-E.); avilamartinezvale@gmail.com (V.Á.-M.); 2 Endocrinology Department, Instiuto Nacional de Cardiología Ignacio Chávez, Juan Badiano No. 1, Col. 4 Sección XVI, México City 14080, Mexico; fuentevilla_alvarez@hotmail.com; 3 Research Direction, Instituto Nacional de Cardiología Ignacio Chávez, Juan Badiano No. 1, Col. Sección XVI, Mexico City 14080, Mexico; gvargas63@yahoo.com; 4 Physiology Department, Instituto Nacional de Cardiología Ignacio Chávez, Juan Badiano No. 1, Col. 4 Sección XVI, México City 14080, Mexico; vcastrejn@yahoo.com.mx; 5 Sub-Directorate of Basic Research, Instituto Nacional de Cardiología Ignacio Chávez, Juan Badiano No. 1, Col. Sección XVI, Mexico City 14080, Mexico; edith.alvarez@cardiologia.org.mx; 6 Computed Tomography Department, Instituto Nacional de Cardiología Ignacio Chávez, Juan Badiano No. 1, Col. Sección XVI, Mexico City 14080, Mexico; reginadelamora@hotmail.com; 7 Cardiovascular Biomedicine, Instituto Nacional de Cardiología Ignacio Chávez, Juan Badiano No. 1, Col. Sección XVI, México City 14080, Mexico; pertorisr@yahoo.com.mx; 8 Cardiovascular Line in American British Cowdray (ABC) Medical Center, Sur 136 No. 116 Col, Las Américas, México City 01120, Mexico

**Keywords:** bicuspid aortic valve, aortic stenosis, miRNAs

## Abstract

Deregulation of micro-RNAs (miRNAs) may contribute to mechanisms of injury in the bicuspid aortic valve (BAV). Our objective was to investigate the expression of miRNAs in aortic tissue from patients who underwent aortic valve replacement for aortic stenosis and its relationship with aortic dilatation. The study included 78 patients, 40 with bicuspid aortic valve (BAV) and 38 with tricuspid aortic valve (TAV). The expression of miRNA-17-5p, hsa-let-7e, and miRNA-196a-5p in human aortic tissue was evaluated by a reverse transcriptase polymerase chain reaction (RT-qPCR). Comparative analysis between patients with BAV and controls with TAV explored the association between the miRNAs and aortic dilatation (AD), calcification, valve dysfunction, and stenosis. The results showed that the expression levels of miRNA-Let-7e-5p and miRNA-196-5p were mostly increased in patients with BAV and aortic dilatation (*p* = 0.01 and *p* = 0.01), respectively. In contrast, the levels of miRNA-17a-5p (*p* < 0.20) were lower but without a statistically significant difference. The downregulation of miRNA-17a-5p and the upregulation of miR-Let-7e-5p and miR-196-5p were related to an increased risk of AD risk. Subjects with BAVs with or without double aortic lesions had higher expression levels of Let-7e-5p and miRNA-17a-5p vs. TAV. In all patients, we found an inverse correlation of MiRNA-196-5p with High-Density Lipoprotein-Cholesterol (HDL-C) and indexed valvular area. In subjects with a higher expression of miRNA196, lower levels of HDL-C correlation (r^2^) [r^2^ 0.27 (*p* = 0.02)] and a lower indexed valvular area [r^2^ 0.28 (*p* = 0.05)] were observed. In the specific analysis for each patient group, it was found that in control subjects with tricuspid aortic valve (TAV), miRNA-196-5p had a positive correlation with valvular calcification (r^2^ = 0.60, *p* = 0.02). Deregulation of miRNAs in the aortic tissue of a BAV may influence valvular stenosis, dysfunction, and concomitant aortic dilation. This information could help to define potential therapeutic target strategies to improve the prognosis and treatment of BAV.

## 1. Introduction

Bicuspid aortic valve (BAV) is a prevalent congenital heart defect and may occur alone or in a syndromic complex [1,2]. The presentation of BAV is heterogeneous and complex, which has led to attempts to classify it by phenotypes [3,4,5]. For clinical, surgical, interventional, and research purposes, an international consensus nomenclature has recently been proposed for congenital BAV and its aortopathy [6]. This nomenclature could help to decipher strategies to evaluate and treat it [7] because it is a common cause of premature death in young people.

Understanding the clinical behavior of patients with BAV is important since this congenital malformation presents in a classic manner and with syndromic states [1,8], all of them complicated with calcification in 58%, aortic stenosis aortic in 83% [9], and coarctation of the aorta in 57.2%, which predominates in very young subjects [10]. Other complications are hypoplasia of the aortic arch in 50.3%, pulmonary hypertension in 39%, aortic valve dysfunction, and infective endocarditis between 10 and 30%, reported in ancient times [11] and currently in ranges from 0.2% to 2.0% [12]. The dilation of the ascending aorta has a prevalence between 33 and 80% [13] and aortic dissection BAV is associated with at least two forms of aortic dilatation [14,15,16]. It has been postulated that the aortic root or annulus dilatation phenotypes and that of the aortic arch or tubular may indicate different genetic origins, although there is no complete evidence of whether this is regulated by other molecules [17] in patients with aortic dilatation or dissection [12]. In subjects with BAV, it has been suggested that epigenetic control may be related to the secondary effect of disrupted aortic flow to the BAV [18,19].

Experimental studies have found differences in the effect of ascending aortic smooth muscle cells derived from neural crest cells on alterations in contraction when compared with a non-BAV control and with cells arising from the paraxial mesoderm of the descending aorta [6]. Experimentally, it has also been seen that neural crest-derived cells, when inhibited with rapamycin, increase the mTOR signaling pathway, which improves contractile function [20] and this makes sense since deregulation of these receptors has also been found [21]. On the other hand, it has been seen that when NOTCH1 is induced there is deficient differentiation into smooth muscle and endothelial cells in human pluripotent stem cells. Therefore, it has been hypothesized that these manifestations may contribute to the development of aortic disease in patients with BAV [22].

Thus, there is evidence of the role that NOTCH1 plays in signaling cell differentiation and apoptosis [23].

Thus, identifying whether miRNAs can act as regulators in certain disease processes will be a first step that will have the subsequent direction of identifying in the future whether they also exert it on NOTCH1 since it has been found that Sirtuin 1 (SirT1) is a protein deacetylase of the sirtuin family, whose activation appears beneficial for cardiac diseases. A recent study has shown that SirT1 can limit Notch signaling in vascular growth model systems. A correlation between increased SirT1 expression and decreased expression of Notch signaling effectors has been found, suggesting that altered interaction between SirT1 and Notch signaling might be involved in BAV pathogenesis and be proposed as potential markers [24].

The underlying pathogenesis of BAV-associated aortopathy involves, in addition to genetic bases, several important mechanisms, such as the hemodynamic forces during the development of aortic dilatation, because abnormal cusp formation during embryogenesis leads to altered flow and shear stress. Therefore, the hypothesis that has been proposed in individuals with BAV is that due to the oscillatory shear (OS) present in the fibrous cap, it stimulates the ECs and this leads to the modification of the mRNA and microRNA (miRNA) within which the disease can be induced [25].

In this regard, miRNAs have been shown to fill the gap between genetic and hemodynamic factors, regulating gene expression on the one hand and responding to environmental changes at the post-transcriptional level [25]. In the structural abnormality of the bicuspid aortic valve, miRNAs are sensitive to the shear stress that occurs in endothelial cells and therefore could participate as a mechanosensitive regulatory factor in some valvular disease conditions [26]. Aortic valve sclerosis and calcification is a specific condition that is related to the differential shear stresses observed during the cardiac cycle. Each side of the valve experiences a distinct shear profile that is sensed by the overlying endothelium. The fibrous layer facing the aorta experiences oscillatory shear stress where genes up- or downregulated by miRNAs as well as paracrine mediators such as BMP4, cathepsin K, and matrix metalloproteinases MMP-2 and MMP-9 may act.

In the endothelial layer, there is a highly inflammatory phenotype where adhesion molecules are expressed among other inflammatory markers [26].

There is accumulating evidence suggesting that altered miRNA expression patterns may modify valvular tissue homeostasis, leading to bicuspid aortic stenosis. Data obtained on miRNA expression levels in isolated endothelial cells and differences between bicuspid aortic valves also suggest that dysregulated reactive oxygen species metabolism leading to apoptosis may also contribute to triggering aortic valve degeneration [27], as they demonstrated a marked increase in miRNAs in BAV leaflets compared to TAV, which was inversely correlated with increased SMAD7 mRNA expression in both human and porcine valve interstitial cells. Furthermore, the authors examined functional changes between BAV and TAV, showing an increased expression of MMP2 and MMP9 in BAV leaflets, indicating a disorder in the extracellular matrix of BAV leaflets, probably inducing valve calcification [28,29]. miRNAs are selectively exported to the extracellular space and incorporated into extracellular vesicles (exosomes, microvesicles) or lipoproteins that protect them from degradation in the extracellular space [30]. The regulation they perform on genes has led to their association with various conditions including cardiovascular diseases, suggesting their potential use as noninvasive biomarkers for diagnosis, prognosis, and treatment [31]. Valvular calcification is evaluated by computed tomography and echocardiography to determine the type of valvular morphological change and the type of aortic stenosis, which may be subvalvular, degenerative supravalvular, rheumatic, or congenital such as BAV, where during embryonic development alterations occur in the morphogenesis of the sigmoid arteries due to the fusion of two free edges and in some cases replacement by a fibrous raphe. Valvular degeneration and calcification occur during the development of the individual into adulthood. In translational research, the combinatorial study of clinical damage attempts to identify miRNA candidates that regulate specific genes in specific conditions of subjects with BAV and TAV to define their expression and determine if they have a role in each specific condition. Therefore, performing a bioinformatic analysis of possible relevant target miRNAs in BAV and TAV could simultaneously determine whether there are miRNAs that modulate calcium metabolism, inflammation, valvular calcification, or dilation in the aorta or inform about the possibility of other convergent pathways [32]. The selected miRNAs for this study were chosen based on their well-documented roles in biological processes relevant to these conditions. Specifically, Let-7e is involved in the regulation of inflammation and vascular remodeling [33], miR-17 plays a critical role in cell proliferation and oxidative stress [34], and miR-196 is associated with development and cell differentiation in cardiovascular tissues [35,36]. This targeted approach helps to focus on key pathways that could link molecular mechanisms with the observed phenotypes in BAV and TAV.

It also establishes a field of research on its participation in cardiovascular pathological processes. We performed a bioinformatics analysis to identify the participation of miRNAs in calcification, aortic dilatation, and coarctation. This analysis showed that the miRNA-17-5p, hsa-let-7e, and miRNA-196-5p could be involved in this process. So, our objective was to evaluate the expression of these miRNAs in subjects with aortic stenosis and dilatation with BAV and TAV in the Mexican population.

## 2. Results

### 2.1. Characteristics of the Study Population

The study included 78 patients, 40 with BAV and 38 with TAV. In the BAV group, 73% (*n* = 29) were male and 23% (*n* = 9) were female, while in the TAV group, 84% (*n* = 32) were male and 21% (*n* = 8) were female, with no statistically significant difference in sex distribution between the groups. However, a significant difference was observed in age, with the BAV group having a mean age of 54 ± 10 years compared to 62 ± 13 years in the TAV group (*p* = 0.007).

The laboratory findings showed lymphocyte counts were significantly higher in the BAV group (median 2.1, interquartile range 1.6–2.6) compared to the TAV group (median 1.5, interquartile range 1.2–2.0) (*p* = 0.01). Additionally, the prevalence of diabetes mellitus (DM) was significantly higher in the BAV group, affecting 23% (*n* = 9) of patients, compared to 7.8% (*n* = 3) in the TAV group (*p* = 0.04). Other hematological and biochemical parameters, such as hemoglobin (HB), total leukocyte count, neutrophils, platelets, total cholesterol, HDL-C, LDL-C, triglycerides, glucose, urea, creatinine, uric acid, erythrocyte sedimentation rate (ESR), and C-reactive protein (CRP), did not show statistically significant differences between the groups (Table 1).

We compared imaging and cardiovascular parameters between patients with BAV and those with TAV. The results revealed significant differences in various clinical and structural aspects between the two groups (Table 2).

Aortic stenosis was found to be significantly more frequent in patients with bicuspid valves, affecting 68% of cases compared to 37% of controls (*p* = 0.01). Although aortic double lesions were also assessed, no significant differences were observed between the groups (45% in bicuspid patients versus 37% in controls, *p* = not significant). Left ventricular ejection fraction (LVEF) was similar in both groups, with an overall range of 15–74%, showing no significant differences between bicuspid and tricuspid patients; also, pulmonary artery systolic pressures (PASPs) did not show.

However, the mean aortic valve gradient was significantly higher in patients with BAV vs. controls with TAV (*p* = 0.0001). Additionally, the aortic valve area and indexed valve area were smaller in BAV compared with the controls (*p* = 0.0001).

Regarding aortic diameter structures, the aortic annulus, sinuses of Valsalva, and sinotubular junction were slightly smaller in patients with BAV vs. controls with TAV, with statistically significant differences (*p* = 0.01, 0.007, and 0.01, respectively) Table 2.

Table 3 shows the comparison between patients with BAV and TAV regarding the type of surgery, aortopathy, and complications. Elective surgery was more common overall, especially in BAV patients, while urgent surgeries were higher in TAV patients. Aortic dissection occurred significantly more in TAV patients, while coarctation was exclusive to BAV. Valvular calcification was observed across both groups. Endocarditis rates were identical, but reinterventions were notably higher in TAV patients. Mortality was present only in the BAV group, though not significantly different.

### 2.2. Surgical Procedure

Table 4 presents the types of surgeries performed, the frequency of each technique, surgical parameters, and the number of fatalities among patients undergoing surgery for aortic stenosis with bicuspid and tricuspid valves.

The Bentall and De Bono procedure was performed on 27 patients (34.6%). The percentage requiring this technique was higher in patients with TAV vs. BAV (*p* = 0.01). A total of 26 patients (33%) underwent aortic valve replacement, with a higher prevalence in the BAV vs. TAV (*p* = 0.008).

Regarding the type of implanted valves, mechanical valves were used in 49 patients (63%), without significant difference between groups, and biological valves were more frequently used in the TAV group vs. BAV (*p* = 0.005).

The median of clamping, cardiopulmonary bypass (CBP), and time was greater in the TAV group (*p* = 0.001) and (0.005), respectively. The bleeding was significantly higher in the TAV vs. BAV (*p* = 0.004). The overall mortality rate was 3 (3.8%), which occurred in the BAV.

Table 5 describes three male patients who died after surgeries related to BAV conditions. All three had systemic arterial hypertension, and their ages ranged from 53 to 64. Comorbidities included prior myocardial infarction for one patient, while the others had no listed conditions. Left ventricular ejection fraction (LVEF) ranged from 46 to 54. The diameters of the aortic annulus, sinus of Valsalva, sinotubular junction, and ascending aorta are listed for each case. Two underwent the Wheat procedure, and one had a Bentall procedure. Two had biological valves, and one had a mechanical valve. All three deaths were due to cardiogenic shock.

### 2.3. miR Expression in BAV

**Figure 1:**
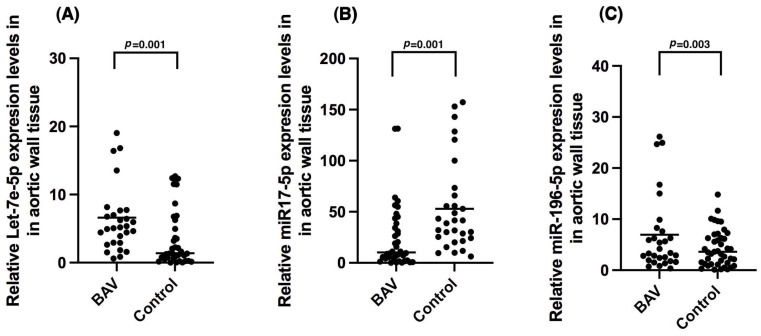
Expression of (**A**) Let-7-5p; (**B**) miR17-5p; and (**C**) miR-196-5p in aortic wall tissue of bicuspid aortic valve (BAV) patients compared to controls. BAV (*n* = 40), control (*n* = 38); value expressed as median. Statistical significance at *p* < 0.05. Statistical test: Mann–Whitney U. Data were normalized with miR-16.

Figure 1 presents three dot plots comparing the relative expression levels of Let-7-5p, miR17-5p, and miR-196-5p in aortic wall tissue between individuals who required aortic surgery with BAV and those who required aortic dilation surgery and had a trileaflet aorta (control group). The comparison was performed using the Mann–Whitney U test according to its nonparametric distribution. A) We show that Let-7-5p expression was significantly increased in the BAV group versus the control group (*p* = 0.001). B) It shows that miR17-5p levels were decreased in the BAV group versus the control (*p* = 0.001). C) The BAV group had an increased miR-196-5p expression versus the control group (*p* = 0.003).

### 2.4. miRs Expression Profiles in Aortic Dilation in Bicuspid Aortic Valve

Table 6 compares the expression levels of Let-7e-5p, miR-17a-5p, and miR-196-5p in aortic wall tissue in individuals with BAV and the controls, with further distinctions based on the presence or absence of aortic dilatation.

The Let-7e-5p expression is notably higher in BAV patients with aortic dilatation (21.58) compared to those without dilatation (5.18) (*p* = 0.02). Additionally, BAV patients with aortic dilatation show significantly higher expression levels than dilated controls (*p* = 0.01). In the case of miR-17a-5p, expression is significantly elevated in BAV patients with dilatation (29.74) compared to those without (7.60) (*p* = 0.01). In controls, individuals with aortic dilatation have a lower expression (40.12) compared to non-dilated controls (120.52) (*p* = 0.02). BAV patients without dilatation also exhibit significantly lower expression than non-dilated controls (*p* = 0.01), though no significant difference is observed between the dilated groups (*p* = 0.20). For miR-196-5p, expression is significantly higher in BAV patients with dilatation (31.10) compared to those without (5.53) (*p* = 0.01). While no significant difference is found between the non-dilated groups (*p* = 0.19), BAV patients with aortic dilatation display significantly higher expression levels compared to dilated controls (*p* = 0.01).

### 2.5. miRs Expression Profiles in Double Aortic Lesion and Stenosis in Patients with Bicuspid and Tricuspid Aortic Valves

**Figure 2:**
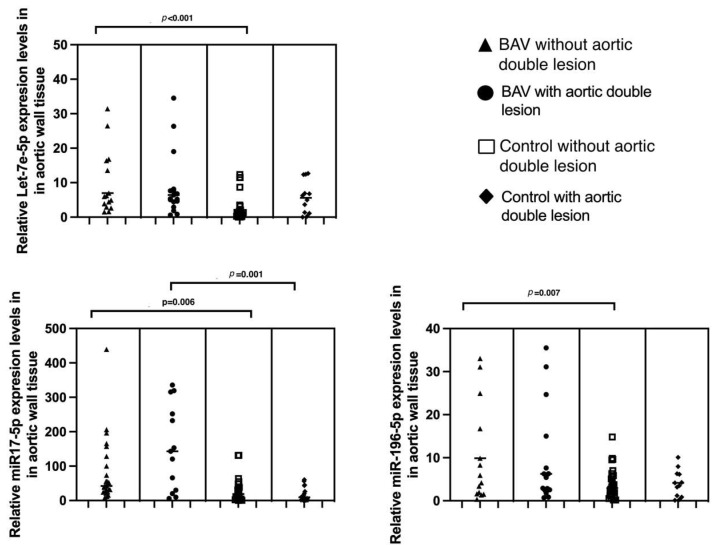
Comparison of expression levels in tissue from control patients and those with a bicuspid aortic value for Let-7e-5p, miR-17a-5p, and miR-196-5p, stratified by aortic double lesion (ADL). BAV with ADL (*n* = 19), BAV without ADL (*n* = 20), control with ADL (*n* = 12), control without ADL (*n* = 26). Values expressed as median. Statistical significance at *p* < 0.05. Statistical test: Mann–Whitney U. Data were normalized with miR-16.

Figure 2 shows three graphs comparing the expression levels of Let-7e-5p, miR-17-5p, and miR-196-5p in aortic wall tissue among four groups. Statistically significant differences are observed between BAV without aortic double lesion and the control without aortic double lesion for all three microRNAs: Let-7e-5p (*p* < 0.001), miR-17-5p (*p* = 0.006), and miR-196-5p (*p* = 0.007), respectively.

Table 7 presents the expression levels of the microRNAs Let-7e-5p, miR-17a-5p, and miR-196-5p across different groups (Control and BAV) with and without stenosis.

Let-7e-5p expression was significantly different in BAV without stenosis (6.61 [1.5–64.05]) and control without stenosis (*p*1 = 0.001). However, no significant differences were found between the groups with stenosis (*p*2 = 0.13).

Regarding miR-17a-5p, BAV without stenosis had levels of 15.10 (0.78–63.99), while those with stenosis were lower at 9.43 (0.09–131.40). In the control group without stenosis, levels were higher at 42.44 (9.58–439.25) and even higher in the control group with stenosis at 142.98 (6.21–335.73). Significant differences were observed between BAV without stenosis and control without stenosis (*p*1 = 0.005), as well as between the groups with stenosis (*p*2 = 0.01).

**Figure 3:**
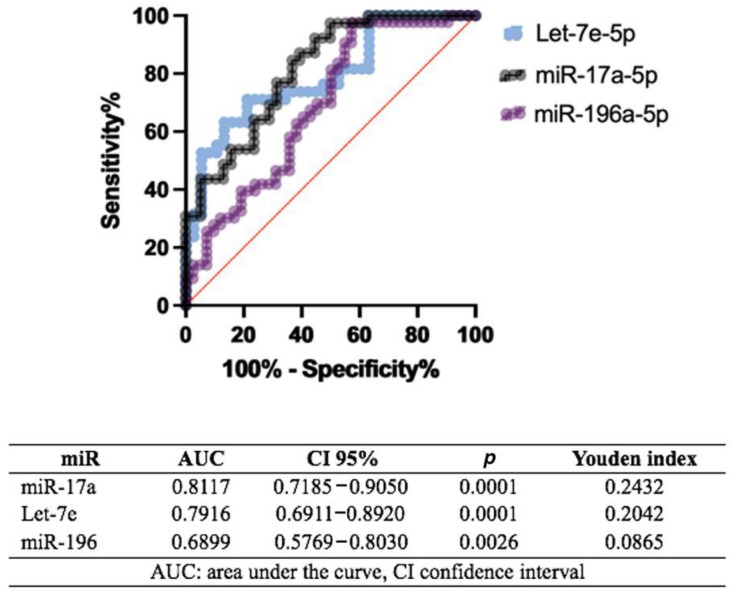
ROC curve analysis. An ROC curve analysis was conducted to distinguish between the control group and individuals with BAV. The entire sample, consisting of 40 BAV cases and 38 controls, was evaluated. The optimal cutoff point was determined using Youden’s index, aiming to maximize the combined sensitivity and specificity.

Figure 3 shows the diagnostic performance of three microRNAs (miRs). miR-17a demonstrates the strongest potential, with an AUC of 0.8117 (95% CI: 0.7185–0.9050) and a significant *p* = 0.0001, yielding a Youden index of 0.2432. Let-7e follows closely, showing an AUC of 0.7916 (95% CI: 0.6911–0.8920) and a *p* = 0.0001, with a Youden index of 0.2042. In contrast, miR-196 has a more moderate AUC of 0.6899 (95% CI: 0.5769–0.8030), a *p* = 0.0026, and a Youden index of 0.0865.

### 2.6. Correlations

In all patients, we found an inverse correlation of Mir-196-5p with HDL-C and with indexed valvular area. In subjects with a higher expression of miR196, we observed lower levels of HDL-C [Corr 0.27 (*p* = 0.02)] and also a lower indexed valvular area [Corr 0.28 (*p* = 0.05)]. In the specific analysis for each group of patients, it was found that in control subjects (TAV) the mirR-196 expression had a positive correlation with valvular calcium of 0.60 (*p* = 0.02), whereas in BAV and TAV, the Mir-196-5p expression with an inverse correlation with a lower valvular area [−0.28 (*p* = 0.05) and −0.56 (*p* = 0.007), respectively] was found.

**Figure 4:**
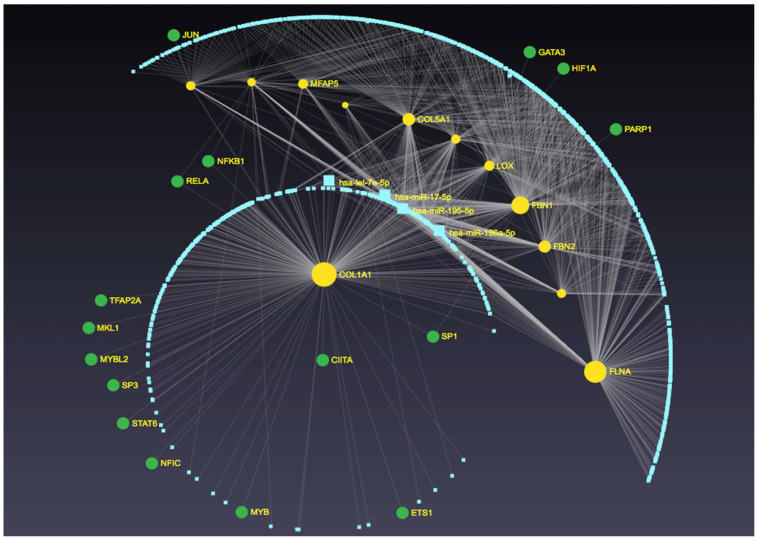
Network visualization of interactions between key microRNAs (Let-7g-5p, miR-17-5p, and miR-195-5p) and their target genes by MiRNet 2.0. Genes associated with aortic damage and extracellular matrix integrity (FBNI, COLSAI, FBN2, MFAPS, LOX, FLNA) are highlighted in yellow, representing the “aortic extracellular matrix and structural integrity” group. In contrast, genes related to inflammatory and transcriptional regulatory pathways (NFKB, JUN, RELA, CITA, SPI, HIFIA, GATA3, PARPI) are shown in green, representing the “inflammatory and transcriptional regulation” group.

In the analysis of the relationship between miRNAs and target genes associated with the development of bicuspid aortic valve (BAV), a set of genes was identified as being regulated by the studied miRNAs (Let-7e-5p, miR-17-5p, and miR-195-5p). Specifically, the miRNAs were found to regulate FBN1, COL5A1, FBN2, MFAP5, LOX, and FLNA, genes that are linked to aortic damage, all highlighted in yellow in Figure 4. These genes are primarily associated with extracellular matrix organization and vascular integrity. In addition, another group of genes, marked in green, was also affected by the miRNAs under study. These genes NFKB, JUN, RELA, CIITA, SP1, HIF1A, GATA3, and PARP1 are mainly involved in inflammatory and transcriptional regulation pathways, including immune response and cellular stress mechanisms. The first group can be referred to as the aortic extracellular matrix and structural integrity genes, while the second group can be termed inflammatory and transcriptional regulation genes, reflecting their roles in relevant biological pathways. (Figure 4).

## 3. Discussion

Ascending aortic aneurysm (AAoA) is considered a high-risk factor for mortality due to its association with a high fatality rate. This is largely because the dilation is often asymptomatic, and the main symptoms tend to appear only when the dilation or dissection has become severe. As a result, the late diagnosis of AAoA can lead to catastrophic outcomes. Dilation has been found in 50–70% of patients with bicuspid aortic valve (BAV) [1,37,38]. Regarding the mechanism of damage, the involvement of epigenetic factors interacting through various cellular signaling pathways has been proposed. Among these mechanisms, DNA methylation, histone modification, and non-coding RNAs (such as microRNAs) are key players [39].

This suggests that valvular and aortic deformation may occur from embryonic development, because endothelial cells have several transitions before reaching a well-differentiated state, which could lead to defects in signaling pathways during the embryonic stage, resulting in the formation of the ascending aorta and the aortic valve. Therefore, a poorly differentiated cellular state could contribute to susceptibility to aneurysm development [40,41].

In BAV, epigenetic mechanisms and those involved in embryonic development could be collaterally regulated by microRNAs (miRNAs) [42] and specific miRNAs could modulate some others in response to hemodynamic changes that arise during mechanical stress in the aorta. All of the above arises from the fact that differential expression of some miRNAs has been found in the bicuspid aortic valve, compared to the tricuspid aortic valve [41,43,44]. The participation of the miRNAs in these phenomena, supports the theory that aortic tissue-specific microRNAs may exert their regulatory effects on aortopathy through their modulatory effect on functional and structural [45].

In this study, the patients with BAV presented a higher frequency of aortic stenosis vs. the control group, so a requirement for valve replacement was a priority with a predominance of valves in young people, a condition that has been described worldwide [46]. The age of the subjects with BAV who required valve replacement ranged between 44 and 64 years, which confirms that the population affected by this pathology differs when compared to populations that have aortic dysfunction due to other causes. Also, we found that there was lower expression of miR-17a-5p in BAV tissue (compared to controls) which might indicate impaired control over calcification pathways [47], since in other studies it has been found that miR-17a-5p regulates genes involved in calcification, such as RUNX2, BMP2, and ANKH [48].

A reduction in miR-17a-5p could lead to enhanced calcification of the aortic valve by failing to suppress these osteogenic pathways [49]. This would contribute to the observed stenosis, particularly in the BAV group. Also, in bicuspid aortic valve dysfunction, the aortic wall has been found to be exposed to high blood flow, in which endothelial and smooth muscle cells are able to sense and respond to changes in flow conditions. The diverse altered morphology of BAV leads to alterations in blood flow dynamics in the valve cusps and aortic wall, which may, in turn, increase the risk of developing calcification [50] aortic stenosis and/or regurgitation, aortic dilatation, and complications such as endocarditis and aortic dissection.

The development in the pathogenesis of the vascular wall has been consistently studied [51]. miRs regulate gene expression by inhibiting their target mRNAs, and some very specific ones regulate the response to hemodynamic changes. In our study, we found that the participation of miRNAs in the aortic damage of patients with BAV is evident, as significant differences were observed in the expression of Let-7e-5p, miR-17-5p, and miR-196-5p in this group when compared to control patients. Regulatory changes involving these miRNAs have already been reported in various cardiovascular conditions, including BAV [32]. Studies have also been reported evaluating the antagomir effect through antioxidant enzymes to influence the reduction in cardiac hypertrophy [52].

The role of miRs in the pathophysiology of BAV and our findings lead us to hypothesize that the differential expression of miRs in BAV patients may indicate a possible dysregulation of gene expression, contributing to the pathological remodeling observed in BAV. Elevated miR-130a expression in plasma was associated with aortic dilatation in children with bicuspid aortic valve (BAV) [53]. There are few studies in this context but they have been proposed as potential future biomarkers [54].

The increased expression of Let-7-5p and miR-196-5p in the BAV group suggests their involvement in abnormal aortic valve tissue development and inflammation [55].

However, it is relevant to mention that in this study we found an inverse correlation of the increased expression of miR-196-5p with lower levels of HDL-C and a positive correlation of miR-196-5p with elevated levels of valvular calcification, which is interesting since an association of miR-196-5p has been found with bone development and osteoblast differentiation, bone mineral density, and fracture risk classification through Genome-Wide Association Studies (GWAS) [56,57], just as it has been found as a possible circulating exosomal biomarker to diagnose postmenopausal osteoporosis, with which we could postulate that it participates in calcium metabolism and with dyslipidemia. It is known that extracellular vesicles (EVs) play a vital role in physiological and pathophysiological processes by transferring microRNAs (miRNAs) to distant tissues and miR-196-5p has been shown to suppress osteoclast-like cell formation and mitochondrial energy metabolism in mouse cells, suggesting that it might be a crucial factor for muscle/bone interaction via EVs [58]. On the other hand, mechanical stress is included among the extrinsic factors that are important for both muscles and bones. The role of mechanical stress in muscle–bone interactions remains unknown; however, it has been found that through small RNA sequencing analysis the effect of fluid flow shear stress (FFSS) elevated the expression of miR196a-5p and miR155-5p with the suppressor actions of osteoclast formation and low expression in the bone cells in a mouse model [59], and this makes sense with what happens in patients with BAV, where blood flow through the aorta is laminar and can cause turbulent transitions, and it has been seen that the global turbulent kinetic energy is higher in subjects with BAV compared to TAV, in addition to the increase in wall shear stress induced by BAV in the upstream direction [60]. The finding in this study of an increase in miR196-5p in patients with BAV could be associated with the regulation of genes involved in dyslipidemia, such as those involved in bone and calcification metabolism, as well as with alterations in FFSS, which would lead to the joint search for the type of regulation that miR-196 exerts on genes related to muscle, bone, and stress flow.

This investigation on miRNA expression in patients with BAV undergoing surgery for aortic stenosis provides information on their role in ascending aorta dilation. The results show a detailed analysis of the clinical characteristics and miRNA expression profiles in these patients. In BAV patients with aortic dilation, the elevated expression of Let-7e-5p could be linked to altered extracellular matrix (ECM) remodeling. Let-7 targets collagen genes and transforming growth factor-beta (TGF-β) signaling, which is central to ECM homeostasis. Dysregulated TGF-β signaling is a hallmark of BAV-associated aortopathies [61].

Likewise, Let-7 miRNAs may contribute to vascular smooth muscle cell (VSMC) dysfunction, promoting abnormal ECM synthesis and aortic wall stiffening, common characteristics in BAV-associated aortic dilation [62]. The let-7 family is the second microRNA found in recent research and is highly expressed in the cardiovascular system. The expression of let-7 in cardiovascular diseases, such as cardiac hypertrophy, cardiac fibrosis, dilated cardiomyopathy (DCM), myocardial infarction (MI), arrhythmia, angiogenesis, atherosclerosis, and hypertension, and the cardiovascular differentiation of embryonic stem cells, deserves a special focus in the damage of congenital diseases, whose onset is embryonic with a varied and catastrophic outcome in the development of the affected individual [63].

The decreased expression of miR-17-5p in BAV patients raises questions about its regulatory role in VSMC proliferation and valve development. miR-17-5p is part of the miR-17-92 cluster [64], which influences cell cycle progression and Notch signaling. Reduced miR-17-5p could affect the expression of NOTCH1, a gene implicated in BAV pathogenesis [28]. Mutations in NOTCH1 are known to predispose individuals to BAV and aortic valve calcification. By downregulating miR-17-5p, there may be increased expression of pro-calcific genes or dysregulated VSMC phenotypic switching, favoring a calcific environment or abnormal vascular remodeling [32]. The elevated levels of miR-196-5p in BAV aortic tissue could be tied to inflammatory processes and ECM degradation [65].

In summary, in patients with BAV, the evolution of the disease begins with the structural change in the valves that leads to complex surgical intervention since it can be associated with aortic dilatation, therefore requiring extensive interventions with techniques such as Bentall and DeBono or Wheat, which increases the risk of mortality in addition to comorbid risks and age. The survival rate found in this series in subjects with BAV was 92.3% at 30 days and 100% in subjects with TAV, which is a finding similar to that found in other series [66]. The same was observed in this series regarding the causes of mortality in adults [67]. However, despite the surgical success that can currently be achieved, all of them occur at the most productive stage of life and it is considered important to evaluate the various pathophysiological mechanisms that participate as well as their regulation, which is why it is vitally important to identify specific antagomirs that affect the various pathways that interact in the structural and functional damage of patients with BAV.

## 4. Materials and Methods

### 4.1. Population

A prospective study in a cohort of patients with BAV was conducted between March 2024 and September 2024. The sample size was calculated, and 64 patients were required to achieve an alpha error of 0.01 and for an alpha error of 0.05. Therefore, 38 patients were required per group. We included 40 cases with BAV and 38 with TAV. The inclusion criteria for patients were women and men who were 18 years old, of Mexican origin with at least two prior generations of Mexican ancestry, confirmed diagnosis of bicuspid and tricuspid valve with aortic stenosis that required aortic and/or aortic valve replacement, and with an aortic valve replacement (AVR) using a biological or mechanical prosthesis. In addition, that they had echocardiography, computed tomography, or magnetic resonance imaging, and signed an informed consent form for the surgery and for the research study. All patients who presented for the first time with aortic stenosis were previously analyzed and discussed in an interdisciplinary meeting of surgeons and the Hear team (HT) and the surgical techniques used and proposed were according to their valvular and aortic complication and according to the final management decision of the HT. We excluded patients with other cardiac interventions in addition to AVR, with significant coronary artery disease, immunodeficiency, and oncological or severe concomitant diseases, such as systemic autoimmune disorders, because these conditions could significantly influence miRNA expression. Patients who received treatments that might alter miRNA profiles, such as immunosuppressive therapies, as well as those with cognitive impairments that limit their understanding of the study’s procedures and requirements, were also excluded. Furthermore, individuals who refused to provide samples or relevant medical information, along with pregnant or breastfeeding women, were excluded due to potential implications for interpreting the results.

### 4.2. Measurement of Aortic Stenosis Echocardiographic Parameters

Patients with aortic stenosis (AS) had been previously evaluated preoperatively by echocardiography where the three main criteria for determining AS were used, namely, peak aortic transvalvular velocity, mean valvular gradient, and calculated aortic valve area (AVA). Two-dimensional transthoracic echocardiographic study, TTE, was performed and evaluated by two expert echocardiographers following the recommendations for cardiac chamber quantification and evaluation of valve prostheses. A Phillips EPIC 7 (Philips, Andover, MA, USA) ultrasound was used with an S5-1 (1–5 MHz) transducer. The effective orifice area (EOA) of the PrAV (aortic valve prosthesis) was calculated employing the continuity equation. The peak velocity (Pvel) and mean gradient (MG) of the PrVA were obtained in a five-chamber projection. The pulmonary artery (PSAP) was calculated by adding the right atrium pressure to the maximum gradient of tricuspid regurgitation.

### 4.3. Measurement of Valvular Calcium

The acquisition protocol for the measurements was performed using simple computed tomography (CT) of the heart with electrocardiographic synchronization (Revolution CT by General Electric with 256 Syngo Via VB60S detectors by Siemens, Bayswater, VIC, Australia). It was a prospective protocol in the diastolic or systolic phase according to the patient’s heart rate, with a thickness of 2.5 mm and an interval of 1 mm. The scanning range goes from the ascending aorta to the diaphragmatic face of the heart. The calcium score is calculated using the Agatston method, which is obtained using post-processing software (Syngo Via, VB40 Siemens Healthineers Forchheim, Germany) for the analysis. Calcification is identified as an attenuation above 130 HU and an area greater than or equal to 1 mm^2^. The Agatston score is obtained by integrating the product of the total plaque area and a cofactor based on the calcium attenuation of the plaque in Hounsfield units (HUs) [68].

### 4.4. Measurement of Aortic Diameter

We obtained aortic size metrics from computed tomography (CT) angiograms. Aortic diameters on baseline CT were obtained from the clinical radiology report by trained technicians using centerline-derived reconstructions. Maximum diameter measurements were also recorded at various aortic locations including the sinuses of Valsalva (cusp-to-cusp technique); sinotubular junction; mid-ascending aorta; proximal arch; mid-arch (immediately distal to the left common carotid artery); 2 cm distal to the left common carotid artery; and 2 cm distal to the left subclavian artery. The maximum diameter in the ascending aorta, including and excluding the aortic sinuses, was also recorded. The centerline length of the ascending aorta (defined as the aortic annulus to the innominate artery) was measured using specialized software (Vitrea, Vital Images Inc, product version 7.14, Minnetonka, MN, USA). Clinical impression of growth had comparative results within the surveillance interval.

Additional suggested metrics for aortic size damage or dilatation to index patient body size suggested within the guiding studies were also calculated using the Aortic Size Index (ASI; cm/m^2^): maximum ascending aorta diameter/body surface area [69], the Aortic Height Index (AHI; cm/m): maximum ascending aorta diameter/patient height; and [70], the aortic cross-sectional area/height.

### 4.5. Ethical Considerations

Ethical approval was obtained on 12 January 2024. The Research and Ethics Committee of our institution approved the research protocol (Institutional protocol number: 24-1431). This study was carried out according to the international ethical standards and the General Health Law, as well as the Helsinki Declaration, modified at the Congress of Tokyo, Japan. An informed consent form written for recruitment and the use of patient data was obtained from each patient, control subject, or their legal representative.

### 4.6. Surgical Technique and Sample Collection

**Figure 5:**
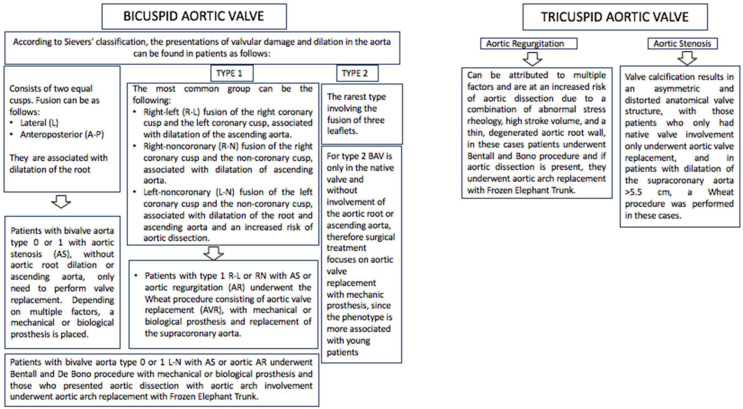
Diagram shows the different BAV phenotypes that exist according to Sievers’ classification. It also explains the conditions that may be associated with them, which leads to the use of different surgical techniques. In this series, only patients with malformations 0 and 1 were treated. There were no patients with BAV type 2 [7].

The surgical technique was selected according to the individual problem of each patient with BAV taking into account the Sievers classification [7]. A diagram showed the procedures and types of techniques performed in this study, and we show the considerations chosen by the Heart team on the type of surgical technique for the treatment of patients in this study. It was based on Sievers’ classification, Figure 5.

After following the anesthesiology protocol and placing the patient under general anesthesia, the sternum and pericardium were opened. Heparin was administered, the patient was cannulated, and cardiopulmonary bypass began. Under mild hypothermia, the aorta was clamped, and antegrade cardioplegia was given. An aortotomy was performed to expose and resect the aortic valve using Metzenbaum scissors and Allis forceps, carefully avoiding damage to the aortic annulus, mitral valve, interventricular septum, and coronary ostia. After valve removal, sutures were placed in the annulus, a prosthetic valve was implanted, and its function was confirmed. Aortic wall samples were collected, the aorta was sutured, the temperature was raised, the aortic clamp was removed, and the patient was weaned off bypass, concluding the procedure. Aortic wall samples were taken during valve replacement surgeries and Bentall, De Bono, and Wheat procedures in both bicuspid and tricuspid valve patients. Small samples were taken in valve replacement cases, while larger samples were collected during annulus and ascending aorta replacements. The samples were rinsed with All-protect Tissue Reagent (QIAGEN, Hilden, Germany, Catalog Number 76405) to safeguard the integrity of the samples and prevent RNA degradation immediately frozen, and stored at −80 °C.

### 4.7. Sample Pulverization

Frozen samples were pulverized using liquid nitrogen to preserve RNA integrity and prevent degradation. This was conducted in a pre-cooled mortar, grinding the tissue into a fine powder. The tissue was then resuspended in Tripure solution (a reagent based on phenol and guanidine thiocyanate) for total RNA extraction. Resuspension followed the manufacturer’s instructions for Tripure™ (Roche Molecular Biochemicals sourced from Basel, Switzerland), ensuring proper tissue homogenization. The resuspended samples were stored at −80 °C until further processing, ensuring RNA stability and preventing degradation.

### 4.8. miRNAs Extraction

miRNAs were extracted from tissue samples using the miRNeasy Tissue/Cells Advanced Kit (Qiagen, Hilden, Germany) according to the manufacturer’s protocol. This kit is specifically designed for efficient isolation of total RNA from tissues, ensuring the integrity and purity of RNA for downstream applications. The extracted RNA was quantified to assess yield and purity and was immediately aliquoted and stored at −80 °C to preserve its integrity until further analysis.

### 4.9. Real-Time PCR

The pulsed reverse transcription reaction was performed to obtain cDNA of Let7a-5p, miRNA17a-5p, and miRNA-196a-5p, with the specific primers for the mature forms, using the TaqMan miRNA RT Kit (TaqMan Advanced miRNA cDNA Synthesis Kit, Applied Biosystem, Foster City, CA, USA, Catalog Number A28007).

Let-7a-5p (ID: hsa-let-7a-5p), miRNA17a-5p (ID: hsa-miR-17-5p), and miRNA-196a-5p (ID: hsa-miR-196a-5p) were quantified using a commercial system kit (TaqMan gene expression assay, Thermo Fisher Scientific, sourced from Waltham, Massachusetts, USA) for microRNA, using the CFX96 real-time PCR system (BioRAD, Hercules, CA, USA). Conditions were 2 min at 50 °C and 10 min at 95 °C, followed by 40 cycles of 15 s at 95 °C and 1 min at 60 °C. Expression levels were measured in duplicate and normalized to the endogenous miRNA-16-5p (ID: hsa-miR-16-5p). The relative quantification was carried out using the following formula: 2^−ΔΔCt^ [71].

### 4.10. Statistical Analysis

Data were analyzed using SPSS software, version 21 (SPSS Inc., Chicago, IL, USA). The normality of each variable was determined using the Kolmogorov–Smirnov test. Quantitative variables were expressed as mean ± standard deviation (SD), while qualitative variables were reported as frequencies with corresponding percentages. Variables were compared using Student’s *t*-test or the Mann–Whitney U test for two groups. Pearson’s correlation analysis was used to assess the association between miRNA expression levels and clinical biochemical parameters. The diagnostic value of miRNA expression was evaluated by calculating the area under the curve (AUC) in ROC models to define the optimal cutoff point.

To create a microRNA (miRNA) interaction network, we focused on three specific miRNAs, let-7e, miR-17, and miR-196, selected for their known or suggested roles in the biological process of interest. Using MiRNet 2.0 [56], a bioinformatics platform developed at the University of Ottawa, we identified their target genes by assessing the alignment between the miRNA sequences and their complementary mRNAs. With this information, we generated a network, where each miRNA served as a node, and its interactions with target genes were shown as connections (edges). Further examination of the network allowed us to detect interaction hubs, key nodes, and functional modules, enhancing our understanding of miRNA-driven gene regulation within the biological context.

## 5. Conclusions

Aortic valve stenosis and calcification are common in BAV, and aortic dilatation is an associated complication leading to mortality in young people. The identification of potential miRNAs regulating the damage mechanisms aims to therapeutically intervene before the invasive method of surgery. Let-7e-5p, miR-17a-5p, and miR-196-5p are increased in BAV subjects with aortic stenosis; there are low levels of miR-17a-5p and elevated expression of miR-196-5p in dilated aortic tissue. MiR196-5p was found elevated in subjects with BAV and elevated calcification and was inversely related to low HDL-C levels, which could be related to dyslipidemia, metabolic processes, chronic inflammation, or oxidative stress deregulation. Flow turbulence conditioned by structural alterations in BAV may also be the basis for future studies in human tissue and serum and determine whether they are also associated with the miRNAs studied. This study demonstrates that the analysis of the expression of specific miRNAs in BAV is related to aortic valve damage in subjects with bicuspid aortic valve and the evident relationship is with dilatation, stenosis, and calcification. We consider that these results observed in tissue should be evaluated in the future with samples obtained from serum as well as on the regulatory effect on specific genes. Even so, these findings could define their potential use as regulators in the mechanisms of initial damage and during the development of the disease and be biomarkers of damage or therapeutic targets.

### Limitations

In this study, bicuspid aortas were not analyzed by imaging to define the structural change in the BAV according to its new nomenclature and classification proposal that delimits them as fused, partially fused, and non-fused, in which it is proposed that each of the malformations can influence therapeutic and research decisions, so future identification by imaging of each BAV phenotype should be considered to establish whether the pathophysiological state is concomitant with the type of hemodynamic flow, as well as perform a stratified analysis of the regulatory participation of the miRNAs. On the other hand, it is interesting with the results of this study to evaluate whether the identified miRNAs exert their regulation on the GATA and NOTCH1 genes involved in the pathogenesis of BAV, which could bring us closer to a precise therapeutic point.

## Figures and Tables

**Table 1:** Distribution of demographic characteristics in relation to cases with bicuspid aorta versus tricuspid aorta.

Demographic	Total (*n* = 78)	Bicuspid Aortic Valve (*n* = 40)	Tricuspid Aortic Valve (*n* = 38)	*p*
Male (%)	61 (78)	29 (73)	32 (84)	0.27
Female (%)	17 (22)	9 (23)	8 (21)	0.54
Age (years)	48 ± 12	54 ± 10	62 ± 13	0.007
BMI (kg/m²)	26 ± 5	26 ± 5	27 ± 4	0.20
Laboratory Analysis Median (Min–Max) Q1, Q2, Q3				
Hemoglobin (g/dL)	14 (8.7–17.8) 12–14–16	12.2 (9.7–17.8) 13.1–14.2–16	14.2 (9.7–17.8) 13.1–14.2–15.7	0.62
Leukocytes (×10^3^/µL)	7.6 (3.5–18.3) 6–7.1–8.5	7.1 (3.5–11.7) 6.3–7.1–8.4	7.1 (3.9–18.3) 5.4–7.1–9.1	0.95
Lymphocytes (×10^3^/µL)	1.7 (0.4–9.3) 1.3–1.7–3.2	2.1 (0.7–4.2) 1.6–2.1–2.6	1.5 (0.4–9.3) 1.2–1.5–2.0	0.001
Neutrophils (×10^3^/µL)	4.2 (1.1–14.1) 3.1–4.2–5.1	4.1 (1.1–7.7) 3.4–4.1–4.7)	4.5 (2.0–14.1) 3.0–4.5–6.8	0.14
Platelets (×10^3^/µL)	200 (86–453) 175–200–247	214 (98–377) 176–214–254	199 (86–453) 168–199–243)	0.49
Total Cholesterol (mg/dL)	143 (46–246) 121–143–178	142 (46–246) 121–142–171	148 (76–218) 119–148–183	0.65
HDL-C (mg/dL)	40 (14.72) 33–40–46	40 (14–61) 33–40–45	42 (19–72) 33–42–51	0.35
LDL-C (mg/dL)	89 (24–190) 69–89–109	89 (26–190) 70–89–107	89 (23–150) 69–89–119	0.70
Triglycerides (mg/dL)	113 (53–245) 84–110–134	113 (55–190) 87–113–132	103 (53–245) 82–103–139	0.81
Glucose (mg/dL)	99 (77–199) 90–99–112	99 (77–189) 89–99–107	101 (79–199) 90–101–119	0.24
Urea (mg/dL)	22 (9.3–277) 16–22–29	21 (9–277) 16–21–29	22 (10–45) 16–22–28	0.77
Creatinine (mg/dL)	1.0 (1.0–6.0) 0.8–1.0–1.3	0.97 (1–6) 0.81–0.97–1.31	1.0 (1–2) 0.80–1.0–1.3	0.85
Uric Acid (mg/dL)	6 (2.3–13.1) 4.6–6–7.3	6 (3.7–10.1) 4.6–6–7.3	6 (2.3–13.1) 4.6–6–7.5	0.77
ESR (mm/h)	5 (1–59) 2.5–5–18	4 (1–6) 4–6–8	6 (2–59) 3–6–23	0.38
CRP (mg/dL)	5 (2–612) 1.3–5–26.4	6.7 (3–312) 1.4–6.7–16.1	4.3 (2–299) 1.2–4.3–72.5	0.83
Comorbidities *n* (%)				
Diabetes Mellitus (%)	12 (15.4)	9 (23)	3 (7.8)	0.11
Hypertension (%)	29 (37.2)	14 (35)	15 (39)	0.81
Dyslipidemia (%)	12 (15.4)	6 (15)	6 (16)	0.58
COPD (%)	6 (7.7)	1 (2.5)	5 (13)	0.10
Previous MI (%)	4 (5.1)	2 (5)	2 (5.1)	0.67

BMI: body mass index; HDL-C: High-Density Lipoprotein-Cholesterol; LD-C: Low-Density Lipoprotein-Cholesterol; ESR: erythrocyte sedimentation rate; CRP: C-reactive protein; COPD: chronic obstructive pulmonary disease; MI: myocardial infarction. g/dL = grams per deciliter; mg/d = milligrams per deciliter; mm/h = millimeter per hour. The results are expressed in medians, minimum and maximum values, and at the bottom in the three quartiles (Q) 1, 2, and 3. Statistical U Mann–Whitney, χ^2^, and Fisher’s exact test.

**Table 2:** Frequency and averages of imaging and cardiovascular parameters between patients with bicuspid and tricuspid aortic valves.

	Total (*n* = 78)	Bicuspid Aortic Valve (*n* = 40)	Tricuspid Aortic Valve (*n* = 38)	*p*
Native valve	74 (95)	36 (90)	38 (100)	0.11
Aortic stenosis	41 (53)	27 (68)	14 (37)	0.01
Aortic double lesion	32 (41)	18 (45)	14 (37)	0.49
Aortic coarctation	2 (2.5)	2 (5)	0 (0)	0.49
LVEF	51 (15–74) 37–51–60	51 (15–74) 37–51–60	52 (20–62) 39–52–58	0.90
PASP	33 (10–98) 27–33–42	34 (10–80) 25–34–43	32 (10–98) 27–31–39	0.37
Mean gradient	31 (2–102)	46.5 (3–102) 12.4–46.5–67.7	7 (2–90) 3–7–40	0.0001
Valve area	0.70 (0.20–4.20) 0.51–0.70–1.20	0.53 (0.20–1.0) 0.49–0.53–0.70	1.3 (0.38–4.9) 0.77–1.3–2.0	0.0001
Indexed valve area	0.40 (0.10–3.75) 0.29–0.40–0.90	0.30 (0.10–0.60) 0.22–0.30–0.40	0.99 (0.20–3.75) 0.50–0.99–1.60	0.0001
Aortic annulus	24 (9–40) 21–24–27	23 (9–34) 20–23–26	25 (18–40) 22–25–28	0.01
Sinuses of Valsalva	36 (17–79) 31–36–42	34 (17–78) 27–33–40	39 (20–79) 33–39–47	0.007
Sinotubular junction	31 (11–85) 27–31–40	29 (11–80) 26–29–38	35 (16–85) 28–35–44	0.02
Ascending aorta	39 (15–100) 34–39–50	36 (15–84) 34–36–43	46 (16–100) 35–46–59	0.01

LVEF: left ventricular ejection fraction; PASP: pulmonary artery systolic pressure. Statistical with U Mann–Whitney and χ^2^ and Fisher’s exact test.

**Table 3:** Frequency of the type of surgery, aortic damage that patients presented in addition to the bicuspid valve, and complications.

	Total n = 78	BAV n = 40	TAV n = 38	*p*
Type of surgery				
Elective surgery	56 (71.7)	31 (79.5)	25 (64.1)	0.20
Urgent surgery	22 (28.2)	8 (20.5)	14 (35.9)	0.20
Aortopathy				
Aortic dissection	21 (26.9)	6 (15.4)	15 (38.5)	0.03
Coarctation of the aorta	2 (2.5)	2 (5.1)	0 (0)	0.49
Valvular calcification	1501 (552–11,347)	552 (0–7712)	2655 (0–11,347)	
Complications				
Endocarditis	4 (5.1)	2 (5.1)	2 (5.1)	1
Reintervention	10 (12.8)	2 (5.1)	8 (20.5)	0.08
Death	3 (3.8)	3 (7.6)	0 (0)	0.24

BAV: bicuspid aortic valve. Statistical with U Mann–Whitney and χ^2^ and Fisher’s exact test.

**Table 4:** Type of surgery performed, frequency of the type of technique, surgical parameters, and number of deaths among patients operated on for aortic stenosis with bicuspid and tricuspid valves.

Type of Surgery	Total (*n* = 78)	Bicuspid Aortic Valve (*n* = 40)	Tricuspid Aortic Valve (*n* = 38)	*p*
Bentall and De Bono	27 (34.6)	9 (22.5)	18 (47.3)	0.01
Bentall + Arch	2 (2.5)	0 (0)	2 (5.2)	NS
Aortic valve replacement	26 (33)	19 (47.5)	7 (18.4)	0.008
Aortic and mitral valve replacement	1 (1.2)	0 (0)	1 (2.6)	NS
Wheat	16 (20.5)	10 (25)	6 (15.7)	NS
Supracoronary replacement	2 (2.5)	0 (0)	2 (5.2)	NS
Arch replacement	1 (1.2)	0 (0)	1 (2.6)	NS
Ascending aorta replacement	1 (1.2)	1 (2.5)	0 (0)	NS
Warden	2 (2.5)	0 (0)	2 (2.6)	NS
Type of Implanted Valve				
Mechanical	49 (63)	28 (70)	21 (55)	NS
Biological	27 (35)	10 (25)	17 (45)	0.005
Surgical Times’ Median (Min–Max) Q1–Q2–Q3				
Clamping	146 (47–305) 97–146–188	114 (47–280) 95–114–168	152 (66–305) 115–152–214	0.001
CPB	183 (71–630) 135–183–288	150 (71–568) 124–150–240	224 (76–230) 153–224–350	0.005
Bleeding	410 (3–4900) 325–420–680	370 (3–790) 230–370–480	490 (190–4900) 360–490–850	0.004

CPB: cardiopulmonary bypass. Statistical with U Mann–Whitney and χ^2^ and Fisher’s exact test. NS: No Significance.

**Table 5:** Number of cases that died, type of surgery performed, and causes of death.

Case BAV	Age	Gender	BMI	Comorbidities	LVEF	AA	SV	STJ	Ao Asc	Type of Surgery	Type of Valve	Cause of Death
1	53	M	31	SAH previous myocardial infarction	46	26	51	38	34	Bentall	Biological	Cardiogenic shock
2	59	M	24	SAH	54	34	78	69	71	Wheat	Biological	Cardiogenic shock
3	64	M	23	None	54	24	46	40	46	Wheat	Mechanics	Cardiogenic shock

BAV: bicuspid aortic valve; BMI: body mass index; LVEFT: left ventricle ejection fraction; AA: aortic annulus; SV: sinus of Valsalva; STJ: sinotubular junction; Asc Ao: ascending aorta; SAH: systemic arterial hypertension; M: male.

**Table 6:** Comparison of Let-7e-5p, miR-17a-5p, and miR-196-5p expression levels in bicuspid aortic valve (BAV) and control groups with and without aortic dilatation.

	BAV		Control		*p*1	*p*2	*p*3	*p*4
	Non-AD *n* = 22	AD *n* = 16	Non-AD *n* = 18	AD *n* = 20				
Let-7e-5p	5.18 (0.62–88.97)	21.58 (1.50–101.89)	1.84 (0.22–12.67)	0.99 (0.12–12.37)	0.02	0.12	0.10	0.01
miR-17a-5p	7.60 (0.0940–131.20)	29.74 (0.78–131.40)	120.52 (9.87–439.25)	40.12 (6.21–232.11)	0.01	0.02	0.01	0.20
miR-196-5p	5.53 (0.35–97.81)	31.10 (1.67–111.75)	3.17 (0.19–80.28)	3.95 (0.11–14.82)	0.01	0.64	0.19	0.01

BAV: bicuspid aortic valve; AD: aortic dilatation; *p*1: BAV non-AD vs. BAV-AD, *p*2: Control non-AD vs. Control AD, *p*3: BAV non-AD vs. Control non-AD, *p*4: BAV AD vs. Control AD. Statistical with U Mann–Whitney.

**Table 7:** Comparison of Let-7e-5p, miR-17a-5p, and miR-196-5p expression levels in bicuspid aortic valve (BAV) and control groups with and without stenosis.

	BAV		Control		*p*1	*p*2
	Non-Stenosis *n* = 22	Stenosis *n* = 16	Non-Stenosis *n* = 18	Stenosis *n* = 20		
Let-7e-5p	6.61 (1.5–64.05)	6.60 (0.62–155.43)	1.17 (0.02–12.37)	5.61 (0.01–12.67)	0.001	0.16
miR-17a-5p	15.10 (0.78–63.99)	9.43 (0.09–131.40)	42.44 (9.58–439.25)	142.98 (6.21–335.73)	0.005	0.01
miR-196–5p	6.24 (1.33–33.03)	6.94 (0.35–111.75)	2.94 (0.17–14.82)	4.14 (0.11–890.28)	0.74	0.10

BAV: bicuspid aortic valve; *p*1: BAV non-stenosis vs. control non-stenosis, *p*2: BAV stenosis vs. control stenosis. Statistical with U Mann–Whitney.

## Data Availability

Due to confidentiality agreements, the data underlying this study are not publicly available. Access to the data can be requested through mesoto50@hotmail.com following their confidentiality protocols.
